# Canine Perineal Hernia Associated with Prostatic Disorders: Is Castration Really Beneficial? A Retrospective Study

**DOI:** 10.3390/ani15091206

**Published:** 2025-04-23

**Authors:** Putinee Sangmanee, Attawit Kovitvadhi, Wijit Sutthiprapa, Piyathip Choochalermporn, Chunsumon Limmanont

**Affiliations:** 1 Theriogenology Center, Kasetsart University Veterinary Teaching Hospital, Faculty of Veterinary Medicine, Kasetsart University, Bangkok 10900, Thailand; putinee.san@ku.th; 2 KU Vet Innova Nutricare Co., Ltd., Faculty of Veterinary Medicine, Kasetsart University, Bangkok 10900, Thailand; fvetawk@ku.ac.th; 3 Operation and Laparoscopic Center, Kasetsart University Veterinary Teaching Hospital, Faculty of Veterinary Medicine, Kasetsart University, Bangkok 10900, Thailand; doctor_gott@yahoo.com; 4 Kasetsart Veterinary Imaging and Radiotherapy Center, Kasetsart University Veterinary Teaching Hospital, Faculty of Veterinary Medicine, Kasetsart University, Bangkok 10900, Thailand; koi30267@gmail.com; 5 Department of Companion Animal Clinical Sciences, Faculty of Veterinary Medicine, Kasetsart University, Bangkok 10900, Thailand

**Keywords:** dog, neutering, paraprostatic cyst, perineal hernia, prostatic abscess, prostatic disease

## Abstract

Prostatic disorders are commonly observed in older, intact male dogs and have been a primary etiological factor in the development of PH. Limited literature exists regarding correcting PH with castration in dogs suffering from prostatic disorders, including the management and long-term clinical outcomes. This retrospective study aimed to describe the management of prostatic disorders and evaluate outcomes and recurrence following perineal herniorrhaphy with castration. A total of 315 intact male dogs that underwent perineal herniorrhaphy associated with prostatic disorders were classified into two groups: a castrated (PHC; *n* = 184) and a non-castrated group (PHNC; *n* = 131). The clinical signs of the PHC group showed significant improvement in the postoperative, short- and long-term follow-up phases (*p* < 0.001). The enlargement of the prostate gland, heterogeneous parenchyma, and intraparenchymal cystic lesions were observed to be less prevalent in the PHC compared to the other group (*p* < 0.001). The incidence of recurrent PH showed no significant differences (*p* = 0.5). In conclusion, castration combined with perineal herniorrhaphy offers significant advantages for improving outcomes and minimizing the recurrence of PH associated with prostatic pathologies following surgery. This is particularly relevant when managing prostatic cysts, including FNA for drainage, cyst or abscess resection, or partial prostatectomy with omentalization during surgery.

## Simple Summary

Prostatic disorders are linked to androgen-dependent hormones, commonly observed in older, intact male dogs. Prostate enlargement or inflammation may lead to tenesmus or stranguria, elevate intra-abdominal or pelvic diaphragm pressure, and increase the risk of perineal hernia (PH). Most studies in the literature suggest performing perineal herniorrhaphy concurrently with castration to eliminate the hormonal influence and decrease the recurrence rate of PH, although some studies are controversial. The underlying cause of PH recurrence in neutered or intact dogs can arise from multiple factors, including surgeon experience, surgical techniques, suture material, previous repairs, and concurrent prostatic disease. This retrospective study aimed to describe the management of prostatic disorders and evaluate the clinical outcomes and recurrence following perineal herniorrhaphy with castration.

## 1. Introduction

The prostate gland, which is the only accessory sex gland in male canines, plays a vital role in producing and secreting prostatic fluid [1,2,3,4,5,6]. Prostatic disorders are associated with androgen-dependent hormones and are commonly observed in older, intact male dogs [1,2,3,4,5,7,8,9]. According to several studies, benign prostatic hyperplasia (BPH) is the most common prostatic disorder, affecting approximately 50%, 80%, and 90% of dogs aged over 4, 6, and 8 years, respectively [3,4,7,9,10,11,12]. Dogs with BPH may be predisposed to the development of prostatitis or the formation of prostatic cysts or abscesses, which may manifest as intraparenchymal or extraparenchymal (paraprostatic) lesions [12,13].

The clinical manifestations of prostatic disorders can vary from asymptomatic to more severe signs including tenesmus, dyschezia, constipation, small or ribbon-like feces, hematuria, dysuria, stranguria, abdominal discomfort, and septicemia [4,7,12,13,14,15]. Perineal hernia (PH) has been reported to be associated with prostatic disorders in older intact male dogs [15,16]. The enlargement or inflammation of the prostate can lead to tenesmus or stranguria, increase intra-abdominal or pelvic diaphragm pressure, and result in instability of the pelvic diaphragm muscles. As a consequence, abdominal organs may protrude into the perineal region [7,15,16,17,18,19,20]. Hormonal influences are likely to assume a principal role in the pathogenesis of PH; however, the underlying etiopathologies remain ambiguous and are multifactorial, involving factors such as constipation, rectal abnormalities, severe cough, neurogenic atrophy of the pelvic diaphragm muscles, and the effects of relaxin [17,19,21,22,23,24]. Surgical correction, also known as perineal herniorrhaphy, is the preferred treatment option for dogs suffering from PH [17,25,26,27]. Most studies in the literature recommend performing perineal herniorrhaphy concurrently with castration to reduce the recurrence rate of PH [17,28,29,30], although some studies controversially suggest that castration is only beneficial for the treatment of testicular or prostatic diseases [23,31].

Castration results in a significant decrease in testosterone and dihydrotestosterone (DHT) concentrations, inducing apoptosis in prostatic glandular cells and reduction of vascularization within the prostate gland [32,33,34], which is beneficial for preventing prostatic diseases and reducing the recurrence of PH after surgery, except in breeding male dogs. In cases of severe prostatitis, large prostatic cysts, or abscesses, castration should be delayed if no antibiotic treatment has been previously administered. Insufficient administration of antibiotics may result in persistent infections, including chronic urinary tract infections (UTIs) or unresolved prostatic abscesses, thereby posing subsequent challenges in medical treatment after castration [12,14,35,36]. Additionally, diagnosing prostatic lesions via ultrasonography in dogs with PH is limited in offering a comprehensive evaluation of the prostate gland due to its displacement into the perineal region or caudal pelvic cavity [37]. Castration accompanied by perineal herniorrhaphy, conducted under these circumstances and lacking a comprehensive perspective on prostatic diseases, could result in a high risk of unresolved prostatic pathologies and may subsequently result in the recurrence of PH following surgery. The neutered dog with persistent prostatitis, large prostatic cysts, or abscesses may potentially cause recurrent PH. These conditions can lead to chronic tenesmus or dyschezia, which increases intra-abdominal and pelvic diaphragm pressure.

However, the recurrence of PH in both neutered and intact dogs can be attributed to various factors, including surgeon experience, surgical techniques, suture material, previous repairs, and concurrent prostatic disease. Some neutered dogs in our clinical practice experienced PH recurrence and unresolved prostatic pathologies. No previous literature has reported on the surgical and medical management of canine PH associated with diagnosed prostatic disorders, as well as the subsequent treatment outcomes. This retrospective study aimed to describe the management of prostatic disorders and the surgical outcomes combined with the castration of intact male dogs suffering from PH associated with prostatic disorders.

## 2. Materials and Methods

### 2.1. Study Design and Case Selection

This retrospective study reviewed intact male dogs that underwent perineal herniorrhaphy and were diagnosed with prostatic disorders at the Kasetsart University Veterinary Teaching Hospital (KUVTH) in Thailand from 2018 to 2023. All dogs in this study were classified into two groups: a castrated group (PHC), which underwent castration in conjunction with perineal herniorrhaphy, and a non-castrated group (PHNC), which underwent perineal herniorrhaphy only.

### 2.2. Inclusion Criteria

The study included intact male dogs at KUVTH who showed signs of perineal swelling and underwent clinical examination to confirm the symptoms via digital rectal examination. Additionally, all patients suffering from PH were identified with prostatic disorders, including BPH, prostatitis, prostatic cysts, and abscesses. Dogs with testicular tumors and cryptorchidism were included in this study. All patients had perineal herniorrhaphy with different surgical techniques used by the surgeons.

### 2.3. Exclusion Criteria

Patients were excluded from the study if the cause of PH was unrelated to prostatic disorders including severe constipation or enteritis, rectal mass, lumbosacral stenosis, cardiac disease, severe coughing, and suspected prostatic tumor.

### 2.4. Data Collection

The data collection included the following: age, breed, body weight (BW), clinical signs, PH content, and previous treatment history. The pre-operative clinical signs were categorized into 4 groups: urogenital systems, gastrointestinal (GI) systems, systemic signs, and other signs. The diagnoses of PH and prostatic disorders were performed through physical examination and digital rectal examination to evaluate perineal content and prostatic disorders. Complete blood count (CBC) and serum chemistry analysis were performed before surgery to assess overall health and identify systemic diseases. Radiographic and ultrasonographic examinations of the caudal abdominal and perineal regions were performed to examine the positioning of abdominal organs and identify prostatic disorders. Additionally, diagnoses of prostatic disorders were based on semen or prostatic fluid analysis, urinalysis, and/or urine culture. The actual prostatic volume (cm^3^) was measured from the prostate ultrasonography and calculated using the following formula [38]:

$$\mathrm{Actual}\;\mathrm{prostatic}\;\mathrm{volume}\;({\mathrm{cm}}^{3})=\frac{{Width}_{\left(cm\right)}\;\times \;{Length}_{\left(cm\right)}\;\times \;{Depth}_{\left(cm\right)}}{2.6}+1.8$$

The prostate gland enlargement in all case studies was compared to the predicted normal prostatic volume (cm^3^), calculated using the following formula [39]:

Normal prostatic volume (cm^3^) = (0.33 × BW_(kg)_) + 3.28

### 2.5. Medical Treatments

Before surgery, any pre-existing health issues were stabilized. Dogs with azotemia or dehydration required fluid therapy. Cystocentesis was performed in the urinary bladder (UB) retroflexion to release urine and reduce urethral pressure. Urinary catheterization was placed in the UB displacement or retroflexion patients until the day of surgery. Antimicrobials were administered based on the drug of choice for prostatitis/UTIs or the results of bacterial culture and drug sensitivity tests from semen, prostatic fluid, and/or urine samples. The finasteride, 5α-reductase inhibitors (Proscar^®^, Merck & Co., Inc., Arecibo, PR, USA or Firide^®^, Siam Bheasach Co., Ltd., Bangkok, Thailand) at the dosage of 0.1–0.5 mg/kg once a day were used for BPH treatment [40].

### 2.6. Surgical Procedure

The anesthetic protocols were carried out by the anesthesiologist, including sedation and anesthetic induction, following endotracheal intubation and inhalation to maintain anesthesia. The prophylaxis antimicrobial and analgesia drugs were routinely administered before surgery. The aseptic surgical field was prepared at the perineal area and the caudal abdomen, fecal evacuation, and a purse-string suture were placed in the anus. Before surgery, urethral catheterization was placed in all patients for identification of the urethra and the prevention of accidental urethral injury during surgery. For castration, a pre-scrotal or caudal scrotal approach [41] was performed before perineal herniorrhaphy. If a testicular tumor was presented, histopathological evaluation was performed. For perineal herniorrhaphy, all dogs were positioned in sternal recumbency with their hindquarters elevated and tails lifted craniodorsally. Surgical procedures were recorded, including rectal diverticulum correction, colopexy, cystopexy, and additional procedures.

### 2.7. Follow-Up Evaluation

Intraoperative and postoperative complications such as wound dehiscence, hematoma/seroma, rectal prolapse, fecal incontinence, urinary incontinence, and sciatic nerve injury were recorded. The follow-up evaluations after surgery from the available medical records were divided into 3 phases: postoperative (1–2 weeks), short-term (1–2 months), and long-term phase (>6 months—the latest examination). The postoperative clinical signs were compared with preoperative signs and the incidence of hernial recurrence with the causes was evaluated.

### 2.8. Statistical Analysis

All statistical analyses were performed using R-statistic under RStudio ver. 2023.06.0+421 with package Rcmdr ver. 2.9-5. The bar chart was performed by Microsoft Excel (Microsoft Office Home and Student 2021). Descriptive statistics were calculated using mean ± standard deviation (SD) with 95% CI for normally distributed data and the median (range) with 95% CI for non-normally distributed data. The initial body weight and age between PHC and PHNC groups were calculated by Student’s *t*-test where these data were accepted on normally distributed data and homogeneity of variance. The differences in discrete or dichotomous variables between groups were analyzed with the chi-square test. Survival analysis was performed using the survival package for Kaplan–Meier survival analysis and survival curve fitting, while the survminer package was utilized to visualize the survival curves. *p* values < 0.05 were considered statistically significant differences.

## 3. Results

### 3.1. Signalment and Pre-Operative Findings

**Figure 1:**
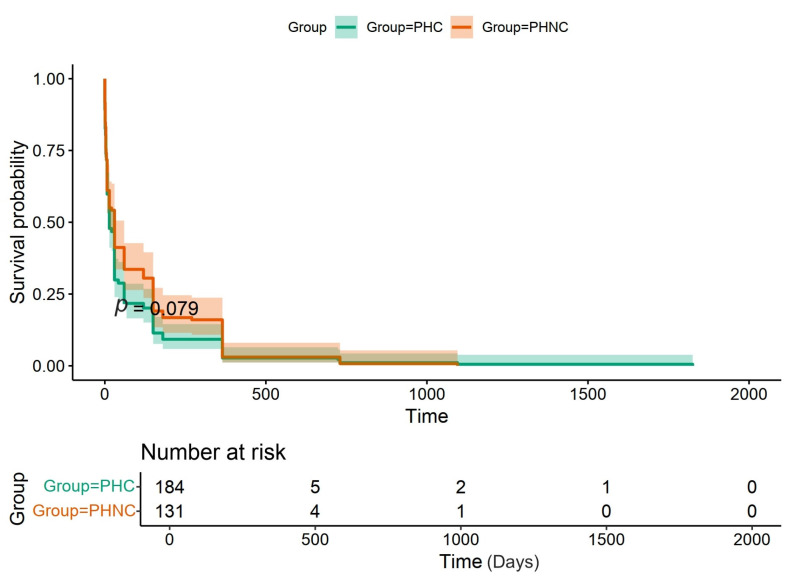
The survival analysis of overall survival time from the duration of clinical signs before being presented to diagnosis in the castrated (PHC) and non-castrated (PHNC) groups with no significant difference between the groups (*p* = 0.079).

During 2018–2023, a total of 332 intact male dogs that underwent perineal herniorrhaphy were evaluated. Of these, 315 cases were in the inclusion criteria associated with prostatic disorders: 184 dogs in the PHC and 131 dogs in the PHNC group. Seventeen cases were excluded due to non-prostatic disorders, including constipation (*n* = 6), cardiorespiratory disease (*n* = 6), trauma (*n* = 3), prostatic carcinoma (*n* = 1), and muscle atrophy (*n* = 1). The most common breeds included Crossbreed (*n* = 78), Chihuahua (*n* = 65), Shih Tzu (*n* = 45), Pomeranian (*n* = 40), Poodle (*n* = 29), along with various Spitz, Siberian Husky, Schnauzer, French Bulldog, Miniature Pinscher, Yorkshire Terrier, Bangkaew, Jack Russell Terrier, Weimaraner, and one each of Affenpinscher, American Pit Bull Terrier, American Toy Fox Terrier, Beagle, Boston Terrier, Coton de Tulear, Italian Greyhound, Labrador Retriever, Maltese, Pekingese, Pug, Thai Ridgeback, and Welsh Terrier. The mean age (±SD; range) was 9 years (±2.7; 3–16; PHC and ±2.5; 4–15; PHNC), while BW were 8.7 kg (±6.6; 2–44 kg; PHC) and 8.3 kg (±6.2; 2–34 kg; PHNC) with no significant difference (*p* = 0.87 and *p* = 0.58, respectively). Pre-operative clinical signs are outlined in Table 1. The most clinical signs were stranguria/dysuria and dyschezia in both groups. The average duration of clinical signs before diagnosis at KUVTH was not significantly different between groups (PHC: 83 days, range 0 day to 5 years and PHNC: 110 days, range 0 day to 3 years) (*p* = 0.079) (Figure 1). BPH from semen analysis was 66.67% (16/24) in the PHC group and 33.33% (1/3) in the PHNC group (*p* = 0.54), while prostatitis was 70.83% (17/24) compared to 66.67% (2/3) (*p* = 1), respectively.

**Figure 2:**
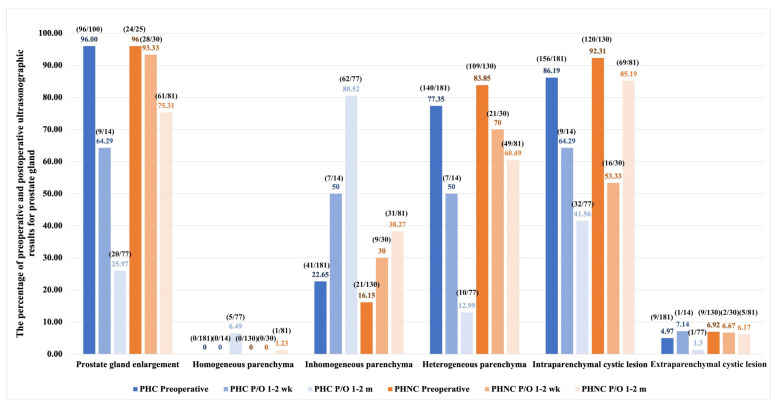
The percentage of preoperative and postoperative (P/O) ultrasonographic results for prostate gland in perineal hernia (PH) dogs associated with prostatic disorders between the castrated (PHC) and non-castrated (PHNC) groups.

Transabdominal and perineal ultrasonography were performed on 181 PHC and 130 PHNC dogs, as shown in Figure 2. Prostate enlargement and intraparenchymal prostatic cystic lesions were the most prominent ultrasonographic findings, with no significant difference between groups (*p* = 1 and *p* = 0.09, respectively). In the PHC group, intraparenchymal cystic lesions were observed in the following sizes: less than 1 cm (61.32%, 111/181), 1–5 cm (24.31%, 44/181), and larger than 5 cm (0.55%, 1/181). In the PHNC group, sizes included less than 1 cm (48.46%, 63/130), 1–5 cm (43.08%, 56/130), and larger than 5 cm (0.77%, 1/130). However, extraparenchymal cystic lesions of the prostate gland showed a significant difference between the groups (*p* = 0.007) and were observed in the following sizes in the PHC group: less than 1 cm (2.76%, 5/181), 1–5 cm (1.66%, 3/181), and larger than 5 cm (0.55%, 1/181). In the PHNC group, lesions were present only in 1–5 cm (6.92%, 9/130). Cystic calculi and sediment were significantly observed in 35.91% (65/181) of the PHC group versus 55.38% (72/130) of the PHNC group (*p* = 0.002). Cystitis/UTIs showed no significant difference (PHC: 84.54% (82/97), PHNC: 85.96% (49/57); χ^2^ = 0.06, *p* = 0.81).

### 3.2. Surgery

**Figure 3:**
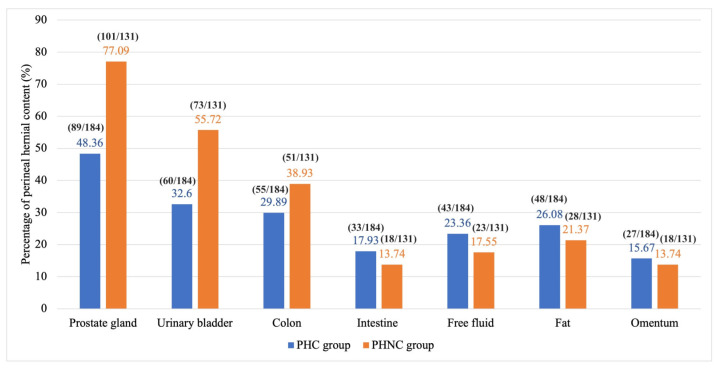
The percentage of perineal hernia (PH) content at the time of surgery between the castrated (PHC) and the non-castrated (PHNC) groups.

**Figure 4:**
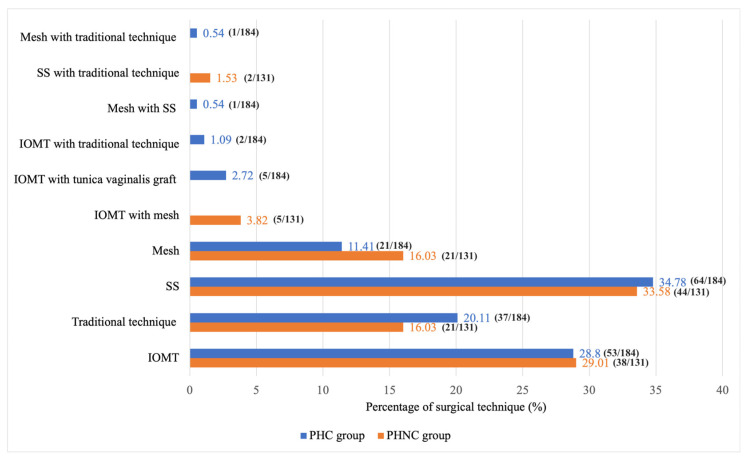
The percentage of surgical techniques for perineal herniorrhaphy between the castrated (PHC) and the non-castrated (PHNC) groups; SS: the sacro-ischial sling method. IOMT: the internal obturator muscle transposition technique.

At the time of surgery, the most herniated contents were the prostate gland and UB in both groups (Figure 3). UB retroflexion was observed in 33.15% (61/184) of the PHC and 38.17% (50/131) of the PHNC group (χ^2^ = 0.84, *p* = 0.36). The most commonly used surgical technique in both groups was the sacro-ischial sling method (SS); 34.78% (64/184) in PHC and 33.58% (44/131) in the PHNC group (Figure 4). The additional intraoperative procedures: 5 PHC dogs and 3 PHNC dogs experienced severe UB necrosis, with 3 PHC dogs showing UB rupture, resulting in partial cystectomy and UB reconstruction, while one dog required UB marsupialization. Additionally, one PHC dog had severe prostatic necrosis, resulting in a total prostatectomy with urethral anastomosis. Cyst wall resection and partial prostatectomy with omentalization were performed for one PHC and three PHNC dogs. Fine needle aspiration (FNA) of prostatic fluid was performed on 13 PHC and 9 PHNC dogs: cysts in 40% (4/10) of PHC and 77.78% (7/9) of PHNC (*p* = 0.17), abscesses in 60% (6/10) versus 22.22% (2/9) (*p* = 0.17). Rectal reconstruction occurred in two dogs from the PHC and PHNC groups due to tears. Three PHC dogs underwent cystopexy and colopexy.

### 3.3. Postoperative Outcome and Follow-Up

**Figure 5:**
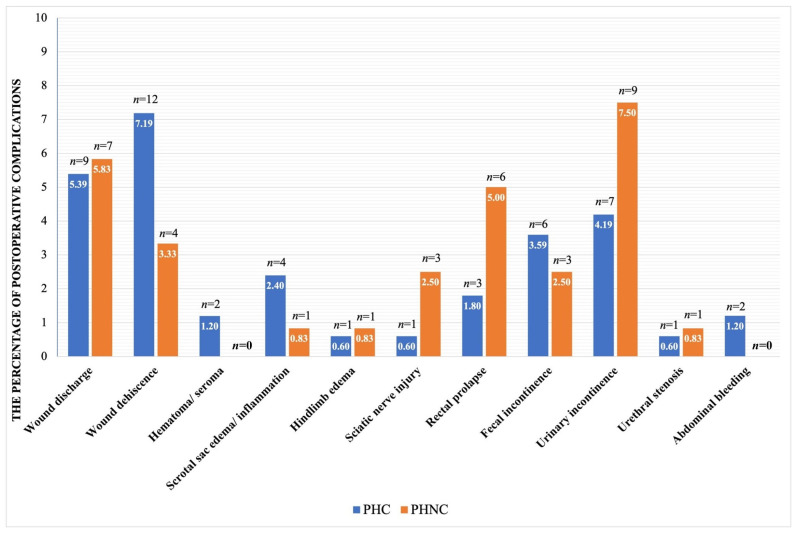
The percentage of postoperative complications between the castrated (PHC) and the non-castrated (PHNC) groups.

After surgery, all dogs were hospitalized to receive IV fluids, analgesics, and antimicrobial treatments. Both groups exhibited surgical wound bruising, inflammation, and mild tenesmus a few days post-surgery. The follow-up assessment included 167 PHC and 120 PHNC dogs, showing no significant differences in postoperative complications between the two groups (χ^2^ = 0.14, *p* = 0.71), as shown in Figure 5. UTIs/cystitis, dyschezia, and tenesmus were significantly more prevalent postoperative abnormalities in the PHNC group throughout all phases of follow-up, as illustrated in Table 2.

#### 3.3.1. Postoperative Phase (1–2 Weeks)

One hundred and sixty-five of the PHC and 115 of the PHNC groups were available for follow-up in this phase. The preoperative clinical signs were significantly improved in the PHC group (PHC: 67.88% (112/165), PHNC: 30.43% (35/115), χ^2^ = 38.10, *p* < 0.001). The incidence of UTIs/cystitis was higher in the PHNC group (64.29% (9/14) vs. 80% (28/35) *p* = 0.29), as shown in Table 2. The prostatic changes observed following surgery are shown in Figure 2. During this phase, the enlargement of the prostate gland was significantly greater in the PHNC group (*p* = 0.025). There were no significant differences in the echogenicity of the prostate gland, as well as in the intraparenchymal and extraparenchymal cystic lesions between the groups (*p* = 0.20, 0.32, and 0.15, respectively). After surgery, three PHC dogs died days later, and one PHNC dog was euthanized due to severe UB necrosis rupture. During this phase, recurrent PH was observed in three dogs in the PHC and PHNC groups.

#### 3.3.2. Short-Term Phase (1–2 Months)

In the short-term follow-up phase involving 104 PHC and 90 PHNC dogs, the pre-operative clinical signs were significantly resolved in the PHC group (PHC: 46.15% (48/104), PHNC 8.89% (8/90); χ^2^ = 32.63, *p* < 0.001). A higher incidence of UTIs/cystitis was still observed in the PHNC group (*p* = 0.004), as illustrated in Table 2. Additionally, prostate gland enlargement, heterogeneous parenchyma, and intraparenchymal cystic lesions were significantly more prevalent in the PHNC group (*p* < 0.001), as shown in Figure 2. Recurrent PH was observed in two PHC and six PHNC dogs.

#### 3.3.3. Long-Term Phase (>6 Months to the Last Examination)

**Figure 6:**
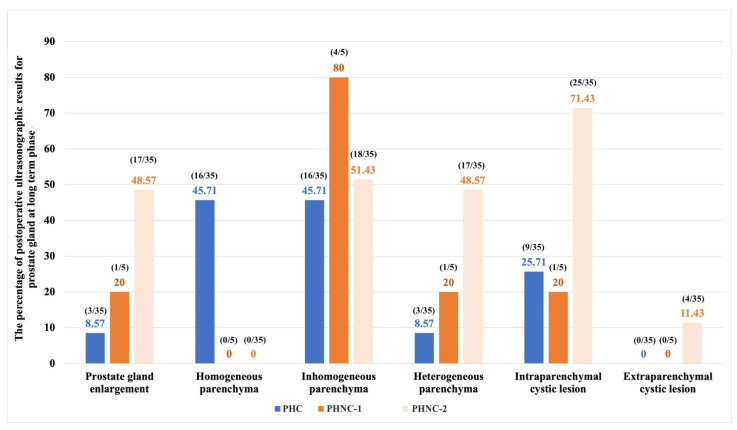
The percentage of postoperative ultrasonographic outcomes for prostate gland in dogs with perineal hernia (PH) associated with prostatic disorders during the long-term phase between the castrated (PHC) and non-castrated (PHNC) groups. These are categorized into two subgroups: castrated within 2 months after perineal herniorrhaphy (PHNC-1) and castration after 2 months of follow-up (PHNC-2).

Seventy-five from PHC and 47 PHNC dogs were available for long-term evaluation and showed highly significantly improved clinical signs in the PHC group (χ^2^ = 13.95, *p* = 0.001). The PHNC group was divided into two subgroups during this phase: seven dogs were castrated within 2 months after perineal herniorrhaphy (PHNC-1), while 40 dogs remained intact or had castration (surgical castration or GnRH implantation) after 2 months of follow-up (PHNC-2). Two dogs of the PHNC-1 continued to exhibit large prostatic cystic lesions and required partial prostatectomy with omentalization during castration. PHNC-2 exhibited significant prostate enlargement (χ^2^ = 148.43, *p* < 0.001), heterogeneous parenchyma (χ^2^ = 29.43, *p* < 0.001), and intraparenchymal cystic lesions (χ^2^ = 22.64, *p* = 0.001), as illustrated in Figure 6.

**Figure 7:**
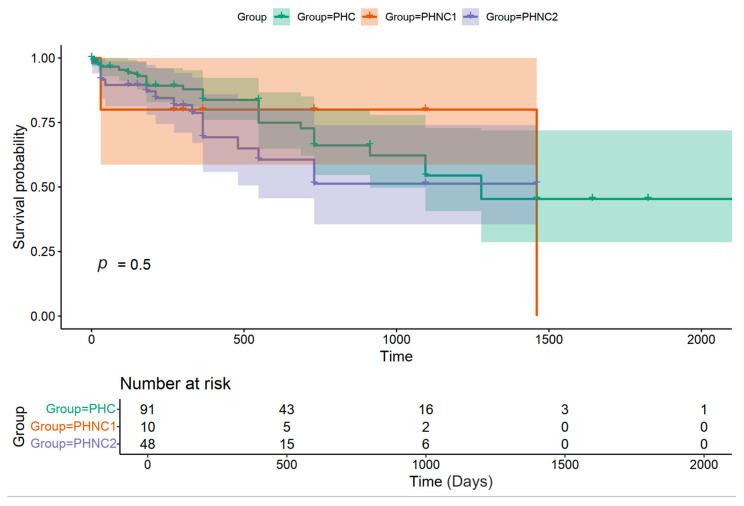
The survival analysis of overall survival time from the duration of no recurrent perineal swelling post-surgery to the last follow-up between the castrated (PHC) and non-castrated (PHNC) groups. These are categorized into two subgroups: castrated within 2 months after perineal herniorrhaphy (PHNC-1) and intact or castration after 2 months of follow-up (PHNC-2) (no significant difference; *p* = 0.5).

The recurrence of PH or rectal pouch did not demonstrate a statistically significant difference among all groups (*p* = 0.5), including PHC: 13.58% (25/184), and PHNC: 16.79% (22/131; with *n* = 4 in the PHNC-1 and *n* = 18 in the PHNC-2). The median duration of no recurrent perineal swelling after surgery to the last visit at KUVTH was 365 days (range: 0 day to 6 years) for the PHC, 730 days (range: 270 days to 3 years) for the PHNC-1, and 365 days (range: 30 days to 4 years) for the PHNC-2 group (Figure 7). There was no significant difference in the PH recurrence rate among the majority of surgical techniques (*p* = 0.15), while the SS method demonstrated a lower recurrence rate compared to the IOMT (SS 29.8% (17/57) vs. 38.10% (16/42)). The following concurrent diseases were presented at the time of PH recurrence in the PHC group: prostatic disorders 12% (*n* = 3), muscle atrophy 8% (*n* = 2), cardiorespiratory diseases 20% (*n* = 5), neurological diseases 8% (*n* = 2), GI diseases with constipation 28% (*n* = 7), trauma 5% (*n* = 1), and unknown cause or loss follow up 20% (*n* = 5). The causes of recurrence in the PHNC-1 group (*n* = 4) include one case each of prostatitis and muscle atrophy associated with coughing, prostatitis, and a paraprostatic abscess. In the PHNC-2 group (*n* = 18), the potential causes of PH recurrence were identified as follows: prostatic disorders accounted for 88.89%, including BPH with prostatitis in 44.44% (*n* = 8); prostatic cysts in 16.67% (*n* = 3, consisting of one intraparenchymal and two paraprostatic cysts); prostatic abscesses in 16.67% (*n* = 3); muscle atrophy with prostatitis in 5.56% (*n* = 1); cardiorespiratory diseases associated with prostatic abscess in 5.56% (*n* = 1); trauma in 5.56% (*n* = 1); and excessive barking in 5.56% (*n* = 1).

## 4. Discussion

The study revealed that prostatic disorders were the primary condition affecting older dogs in both the PHC and PHNC groups, significantly contributing to the development of PH, consistent with previous research findings [20,26,28,42]. In contrast to the literature, the data findings indicate a higher prevalence of dogs with PH among smaller breeds [17,43], which likely reflects the larger population of small-breed dogs in our country. The incidence rate of PH in intact male dogs has been reported to range from 10% to 80% [20,25,37,44,45]. However, the current study shows a higher incidence of prostatic disorders at 94.88% compared to earlier findings. This result was attributed to comprehensive andrological examinations conducted in nearly all cases of PH, including prostate ultrasonography, prostatic fluid/semen cytology and culture. Consequently, the detection of prostatic diseases was higher than in earlier studies. According to the presented data, intact older male dogs of any breed and size with PH should be regularly monitored for prostatic disorders and undergo routine andrological examinations before making decisions regarding the correction of PH. The most common pathological changes in prostate observed through ultrasonography in this study included heterogeneous parenchyma and small intraparenchymal prostatic cystic lesions. According to previous literature, Haverkamp et al. (2019) reported that 84.8% of prostatic cysts were identified through CT scans in dogs older than four years [46], while Ahlberg et al. (2022) indicated that dogs presented with 97.8% of heterogeneous prostate and 93.5% of intraprostatic cysts in cases diagnosed with PH [37]. In the surgical field, the most common hernial contents in both groups were the prostate gland and UB, which was consistent with previous studies [20,25,37]. These results confirm the earlier theory that prostatic disorders primarily cause PH by increasing abdominal and pelvic diaphragm pressure, which may lead to tenesmus and caudal displacement of organs into a hernia. Nevertheless, the etiology of PH may be multifactorial contributors, including rectal abnormalities, neurological factors, trauma, constipation, and severe cardiorespiratory diseases; consequently, additional causes should be ruled out [17,25,37]. The formation of prostatic cysts can result from the obstruction of prostatic fluid in the canaliculi due to BPH, resulting in cysts that vary in size from small to larger cystic lesions [4]. Furthermore, the abnormal positioning of the UB and prostate gland can contribute to obstructive prostatic fluid drainage into the urethra, resulting in cystic formation within the prostate [4,7,37,47].

According to previous reports, the most common signs of PH in dogs include perineal swelling, dyschezia, tenesmus, cystitis, and UB retroflexion [20,24,26,28,42]. From the study results, UB retroflexion has a high incidence (33–38%) in PH dogs, which is a higher prevalence than in previous studies [37]. This condition can lead to urinary tract obstruction, azotemia, and bladder necrosis, resulting in a high rate of postoperative complications and potentially causing death. Urinary catheterization should be performed until surgical intervention is deemed necessary as part of preoperative management to prevent urinary obstruction. Abdominal and perineal ultrasonography serve as effective diagnostic instruments for the evaluation of dogs with PH in conjunction with prostate disorders. These modalities facilitate the evaluation of prostate size, characteristics of the prostate, as well as prostatic lesions, and the localization of abdominal organs [10,20,37,48]. In our report, the PHNC group demonstrated a lower prevalence of prostatic hyperplasia compared to the PHC group, primarily attributable to the limited capability of ultrasonography in fully assessing prostatic characteristics in cases of UB or prostate displacement into the pelvic canal. The CT scan has been reported to assist in evaluating prostatic disorders associated with PH [37,46]; however, this method is not practical in clinical practice due to its high cost and the requirement for anesthesia. The diagnosis of prostatic disorders was based on the cytological examination of semen or prostatic fluid and/or culture [10,13,14]. However, specimen collection was available in some dogs in our study; pain or discomfort is primarily caused by unsuccessful semen collection. Based on the literature, the presence of heterogeneous parenchyma with or without hypoechoic intraparenchymal prostatic cyst lesions from ultrasonography may be assumed as prostatitis [14,37,49]. According to the prior study, prostatitis concurrent with cystitis constituted the most prevalent condition observed in intact male dogs [50]. In our study, UTIs were confirmed through urine cultures in 47% of the PHC group and 69% of the PHNC group, respectively.

For the management of PH associated with prostatic disorders, castration accompanied by the correction of PH is advised for canines exhibiting both conditions simultaneously, as indicated in the majority of the literature [4,15,19]. One month after surgery and during the long-term evaluation, the pre-operative clinical signs showed a more significant resolution in the PHC group compared to the PHNC group. The incidence of prostatic gland enlargement, heterogeneous parenchyma, and intraparenchymal cystic lesions of prostate was lower in the PHC compared to the PHNC and PHNC-2 groups. Furthermore, the PHC group did not show any prostatic abscesses at six months after surgery, in contrast to the PHNC group. Conversely, for dogs with large prostatic cavities, castration should not be performed without drainage or surgical treatment of prostatic cystic lesions [7,51]. The remaining prostatic cystic lesions that develop into a prostatic abscess following castration may result in a recurrence of PH [7,14,35]. In this study, FNA for drainage, cyst or abscess resection, or partial prostatectomy with omentalization was performed as a treatment for large prostatic cysts or abscesses during perineal herniorrhaphy to prevent recurrence of prostatic cystic lesions after surgery in both groups. During the follow-up period, the PHNC group exhibited a recurrence of a prostatic abscess, necessitating multiple drainage procedures, which was not observed in the PHC group. In the PHNC-1 group, two dogs persisted in displaying significant prostatic cystic lesions, requiring a second surgical intervention for partial prostatectomy with omentalization and castration. Furthermore, no recurrence of PH was observed during long-term follow-up. This was attributed to the effective elimination of hormonal effects on the prostate and cystic formation due to castration combined with prostatic cyst management [7,14]. In long-term follow-up, the PHNC group was divided into PHNC-1 (castrated within 2 months after surgery) and PHNC-2 (intact or castrated later). This division results from the veterinarian performing castration once the prostatitis has improved, which may influence the clinical outcomes.

This highlights that castration offers advantages for prostatic atrophy by decreasing prostatic size and limiting bacterial infections, particularly when it is combined with the management of prostatic cysts such as partial prostatectomy or omentalization or FNA of cystic lesions during the initial surgical procedure. Consequently, this approach contributes to reducing recurrent PH associated with prostatitis diseases [15,37]. In this study, caudal scrotal castration can be performed during perineal herniorrhaphy, thereby reducing anesthetic time and eliminating the necessity to change position during surgery [41]. Furthermore, chemical castration or GnRH implantation is recommended for canine patients with systemic health concerns, making anesthesia inadvisable. However, the flare-up effect has been observed in some animals following implantation, potentially worsening the clinical symptoms associated with prostatic disease conditions [4,13,52]. Castrated dogs demonstrated improved clinical signs related to long-term outcomes after castration combined with perineal herniorrhaphy; however, the incidence of perineal hernias differed from that of many other studies [17,19,20,24,41,44,53]. Nevertheless, the recurrence of perineal hernias is influenced by multiple factors, including the experience of the surgeon, various surgical techniques for perineal repair, the type of suture material used, the severity of clinical signs before surgery, and any previous surgical repairs. These elements can contribute to recurrence, as indicated in this study. The surgical repair of PH involves various techniques studied in this research that may impact postoperative outcomes or the recurrence of the condition after surgery; however, the recurrence of PH showed no differences among the surgical techniques. The sacro-ischial sling method was the most common surgical technique used in both groups, which represents an innovative approach to the correction of PH [54], exhibiting minimal complications and a lower incidence of PH recurrence than that of the standardized technique in this study. Nevertheless, the etiology of recurrent PH associated with prostatic abnormalities was diminished in castrated (PHC) dogs. A total of three dogs in the PHC group were suspected to have prostate disease as the cause of the recurrence of PH. One dog had fecal incontinence following surgery and persistent chronic prostatitis. Another dog had an FNA prostatic abscess at the time of surgery and also suffered from persistent chronic prostatitis. A third dog presented with chronic prostatitis and prostate enlargement. Prostatitis appears to disrupt apoptosis in the prostate, and resolving prostate pathologies may contribute to the recurrence of PH.

Postoperative complications were mildly observed a few days following surgical herniorrhaphy in all cases, including wound inflammation, wound dehiscence, scrotal inflammation, hematoma/seroma, and hind limb edema. In the PHC group, three dogs died under the following circumstances: one dog had urethral stenosis, disseminated intravascular coagulation (DIC), and abdominal bleeding after surgery; one dog suffered from pancreatitis and a seizure; and one dog developed progressive azotemia after returning home. The severity of clinical signs may significantly affect the postoperative outcomes of both groups. Outstanding postoperative care in the hospital is recommended, as all patients demonstrated clinical improvement signs. Other than that, one dog in the PHNC group was euthanized after surgery because the owner declined treatment with UB and prostatic necrosis, and the dog developed prostatic necrosis and UB rupture 2 weeks after surgical repair. Additionally, UTIs/cystitis and cystic calculi or sediment were still present in the long-term follow-up of both groups. UTIs or bacterial cystitis are frequently reported in conjunction with prostatitis [14,49,55]. Numerous research studies recommend the administration of antimicrobials for a minimum duration of 4 to 6 weeks for the treatment of cystitis and prostatitis; in certain cases, this duration may extend beyond six months [12,14]. Furthermore, the ongoing evaluation of both prostatitis and cystitis remains essential following surgical procedures [14,49].

The primary limitation of this retrospective study lies in the reliance on medical records, which may lack complete data, including detailed follow-up information and consistent diagnostic methods across cases. Additionally, the decision to perform castration and various surgical techniques to repair PH could potentially introduce variability in treatment outcomes. Another limitation is the relatively small sample size for certain subgroups, which restricts the ability to generalize findings to broader populations. Further research should focus on long-term follow-ups with larger sample sizes to evaluate the recurrence rates and explore alternative surgical and medical protocols. Advanced imaging techniques, like CT or MRI, could provide deeper insights into the pathophysiology of prostatic lesions and PH. Additionally, prospective studies assessing the efficacy of various treatment combinations, including targeted antibiotic therapies and innovative surgical techniques, may help refine the current standards of care. Finally, examining the genetic predispositions in specific breeds could aid in preventive strategies.

## 5. Conclusions

In conclusion, castration in conjunction with perineal herniorrhaphy, presents advantages for enhancing outcomes in the postoperative phase, short-term and long-term, following surgery. This is particularly relevant when combined with the management of prostatic cysts, which may include FNA for drainage, cyst or abscess resection, or partial prostatectomy with omentalization during the initial surgical procedure. Although castration does not influence the incidence of PH recurrence, the recurrence attributable to prostatic pathologies was substantially reduced.

## Figures and Tables

**Table 1:** The percentage of pre-operative clinical signs from 315 perineal hernia (PH) dogs associated with prostatic disorders between the castrated (PHC) and the non-castrated (PHNC) groups.

Pre-Operative Clinical Signs	PHC% (*n* = 184)	PHNC% (*n* = 131)
**Urogenital signs**		
Pollakiuria	2.71% (*n* = 5)	3.81% (*n* = 5)
Stranguria/dysuria	14.13% (*n* = 26)	32.82% (*n* = 43)
Hematuria	4.89% (*n* = 9)	4.58% (*n* = 6)
Urinary incontinence	7.60% (*n* = 14)	7.63% (*n* = 10)
Bloody preputial discharge	1.08% (*n* = 2)	0.76% (*n* = 1)
Testis/scrotal swelling	1.63% (*n* = 3)	0% (*n* = 0)
**Gastrointestinal signs**		
Anorexia	13.58% (*n* = 25)	17.55% (*n* = 23)
Vomiting	14.13% (*n* = 26)	9.16% (*n* = 12)
Diarrhea	14.67% (*n* = 27)	5.34% (*n* = 7)
Dyschezia	64.67% (*n* = 119)	72.51% (*n* = 95)
Tenesmus	52.71% (*n* = 97)	50.38% (*n* = 66)
Constipation	9.23% (*n* = 17)	5.34% (*n* = 7)
Small or ribbon-like-shaped feces	14.67% (*n* = 27)	6.10% (*n* = 8)
Hematochezia	10.32% (*n* = 19)	9.92% (*n* = 13)
Fecal incontinence	0% (*n* = 0)	0.76% (*n* = 1)
Rectal prolapse	1.63% (*n* = 3)	0% (*n* = 0)
Rectal tear	1.08% (*n* = 2)	0.76% (*n* = 1)
**Systemic signs**		
Sepsis	0.54% (*n* = 1)	0% (*n* = 0)
DIC	0% (*n* = 0)	0% (*n* = 0)
Azotemia	18.47% (*n* = 34)	21.37% (*n* = 28)
**Other**		
Perineal swelling	100% (*n* = 184)	100% (*n* = 131)
Perineal necrosis, severe inflammation	9.23% (*n* = 17)	8.39% (*n* = 11)
Perineal rupture	2.17% (*n* = 4)	0.76% (*n* = 1)
Hindlimb lameness	1.08% (*n* = 2)	0.76% (*n* = 1)

**Table 2:** The postoperative outcome, complications, and follow-up between the castrated (PHC) and non-castrated (PHNC) groups are categorized into two subgroups: castrated within 2 months after perineal herniorrhaphy (PHNC-1) and intact or castration after 2 months of follow-up (PHNC-2).

Post-Operative Outcome	1–2 Weeks		1–2 Months		>6 Months		
	PHC% (*n*/Total)	PHNC% (*n*/Total)	PHC% (*n*/Total)	PHNC% (*n*/Total)	PHC% (*n*/Total)	PHNC-1% (*n*/Total)	PHNC-2% (*n*/Total)
**Urogenital system**							
Pollakiuria	0 (0/165)	0 (0/115)	0 (0/103)	0 (0/90)	0 (0/75)	0 (0/7)	0 (0/40)
Stranguria/dysuria	0.60 (1/165)	0.86 (1/115)	0 (0/103)	1.11 (1/90)	1.33 (1/75)	14.29 (1/7)	2.50 (1/40)
Hematuria	0.60 (1/165)	0.86 (1/115)	0 (0/103)	1.11 (1/90)	1.33 (1/75)	0 (0/7)	2.50 (1/40)
Urinary incontinence	4.24 (7/165)	7.82 (9/115)	1.94 (2/103)	6.66 (6/90)	6.66 (5/75)	14.29 (1/7)	7.50 (3/40)
Bloody preputial discharge	0 (0/165)	0 (0/115)	0 (0/103)	0 (0/90)	0 (0/75)	0 (0/7)	0 (0/40)
Testis/scrotal swelling	1.21 (2/165)	1.74 (2/115)	0.97 (1/103)	0 (0/90)	0 (0/75)	0 (0/7)	0 (0/40)
UTIs/cystitis	64.29 (9/14)	80 (28/35)	19.48 (15/77)	41.98 (34/81)	31.43 (11/35)	20 (1/5)	28.57 (10/35)
Cystic calculi/sediment	28.57 (4/14)	26.67 (8/30)	12.99 (10/77)	29.63 (24/81)	22.86 (8/35)	40 (2/5)	34.29 (12/35)
Ureter dilate	0 (0/14)	6.67 (2/30)	0 (0/77)	1.23 (1/81)	0 (0/35)	0 (0/5)	0 (0/35)
Prostatic urethral dilate	0 (0/14)	0 (0/30)	3.90 (3/77)	0 (0/81)	0 (0/35)	0 (0/5)	0 (0/35)
Hydronephrosis	0.60 (1/14)	0 (0/30)	1.30 (1/77)	0 (0/81)	2.86 (1/35)	0 (0/5)	0 (0/35)
Urethral rupture	0 (0/165)	0 (0/115)	0 (0/103)	1.11 (1/90)	0 (0/75)	0 (0/7)	0 (0/40)
UB rupture	0 (0/165)	0.86 (1/115)	0 (0/103)	0 (0/90)	0 (0/75)	0 (0/7)	0 (0/40)
**Gastrointestinal system**							
Anorexia	2.42 (4/165)	2.60 (3/115)	0.97 (1/103)	2.22 (2/90)	4 (3/75)	0 (0/7)	0 (0/40)
Vomiting	3.03 (5/165)	2.60 (3/115)	0.97 (1/103)	0 (0/90)	0 (0/75)	0 (0/7)	0 (0/40)
Diarrhea	3.63 (6/165)	3.47 (4/115)	0 (0/103)	2.22 (2/90)	0 (0/75)	0 (0/7)	2.12 (1/40)
Dyschezia	0.60 (1/165)	4.35 (5/115)	0.97 (1/103)	1.11 (1/90)	5.33 (4/75)	0 (0/7)	6.38 (3/40)
Tenesmus	3.03 (5/165)	6.96 (8/115)	1.94 (2/103)	5.56 (5/90)	4 (3/75)	0 (0/7)	6.38 (3/40)
Constipation	0 (0/165)	0 (0/115)	0.97 (1/103)	0 (0/90)	2.67 (2/75)	0 (0/7)	0 (0/40)
Small or ribbon-like-shaped feces	0 (0/165)	0.86 (1/115)	0.97 (1/103)	0 (0/90)	0 (0/75)	0 (0/7)	2.12 (1/40)
Hematochezia	1.81 (3/165)	0.86 (1/115)	0 (0/103)	1.11 (1/90)	0 (0/75)	0 (0/7)	0 (0/40)
Fecal incontinence	3.63 (6/165)	2.60 (3/115)	0.97 (1/103)	4.44 (4/90)	1.33 (1/75)	0 (0/7)	2.12 (1/40)
Rectal prolapse	1.81 (3/165)	5.21 (6/115)	0.97 (1/103)	0 (0/90)	1.33 (1/75)	0 (0/7)	0 (0/40)
Rectal tear	0 (0/165)	0 (0/115)	0 (0/103)	0 (0/90)	0 (0/75)	0 (0/7)	0 (0/40)
**Systemic signs**							
Sepsis	0.60 (1/165)	0.86 (1/115)	0 (0/103)	0 (0/90)	0 (0/75)	0 (0/7)	0 (0/40)
DIC	0.60 (1/165)	0 (0/115)	0 (0/103)	0 (0/90)	0 (0/75)	0 (0/7)	0 (0/40)
Azotemia	1.81 (3/165)	2.60 (3/115)	4.85 (5/103)	2.22 (2/90)	5.33 (4/75)	0 (0/7)	4.25 (2/40)
Pancreatitis	0.60 (1/165)	1.73 (2/115)	0 (0/103)	0 (0/90)	0 (0/75)	0 (0/7)	0 (0/40)

## Data Availability

Data are contained within the article and Supplementary Materials.

## Supplementary Materials

The following supporting information can be downloaded at: https://www.mdpi.com/article/10.3390/ani15091206/s1, Figure S1: The recurrence rate associated with surgical techniques; Table S1: The recurrence rate associated with surgical techniques.

## Abbreviations

The following abbreviations are used in this manuscript:

BPH	Benign prostatic hyperplasia
PH	Perineal hernia
UTIs	Urinary tract infections
BW	Body weight
GI	Gastrointestinal tract
CBC	Complete blood count
cm^3^	Cubic centimeter
UB	Urinary bladder
SD	Standard deviation
PHC	Castrated group which underwent castration in conjunction with perineal herniorrhaphy
PHNC	Non-castrated group which underwent perineal herniorrhaphy only
kg	Kilogram
cm	Centimeter
P/O	Postoperative
SS	The sacro-ischial sling method
IOMT	Internal obturator muscle transposition technique
FNA	Fine needle aspiration
PHNC-1	PHNC group which castrated within 2 months after perineal herniorrhaphy
PHNC-2	PHNC group which remained intact or castrated 2 months after perineal herniorrhaphy
GnRH	Gonadotropin-releasing hormone
DIC	Disseminated intravascular coagulation
CT scan	Computerized tomography scan
MRI	Magnetic resonance imaging
