# Supplementary figures and images for: Canine Perineal Hernia Associated with Prostatic Disorders: Is Castration Really Beneficial? A Retrospective Study
Source: Animals (Basel). 2025 Apr 23;15(9):1206. doi: 10.3390/ani15091206 (PMC12071062; doi:10.3390/ani15091206)

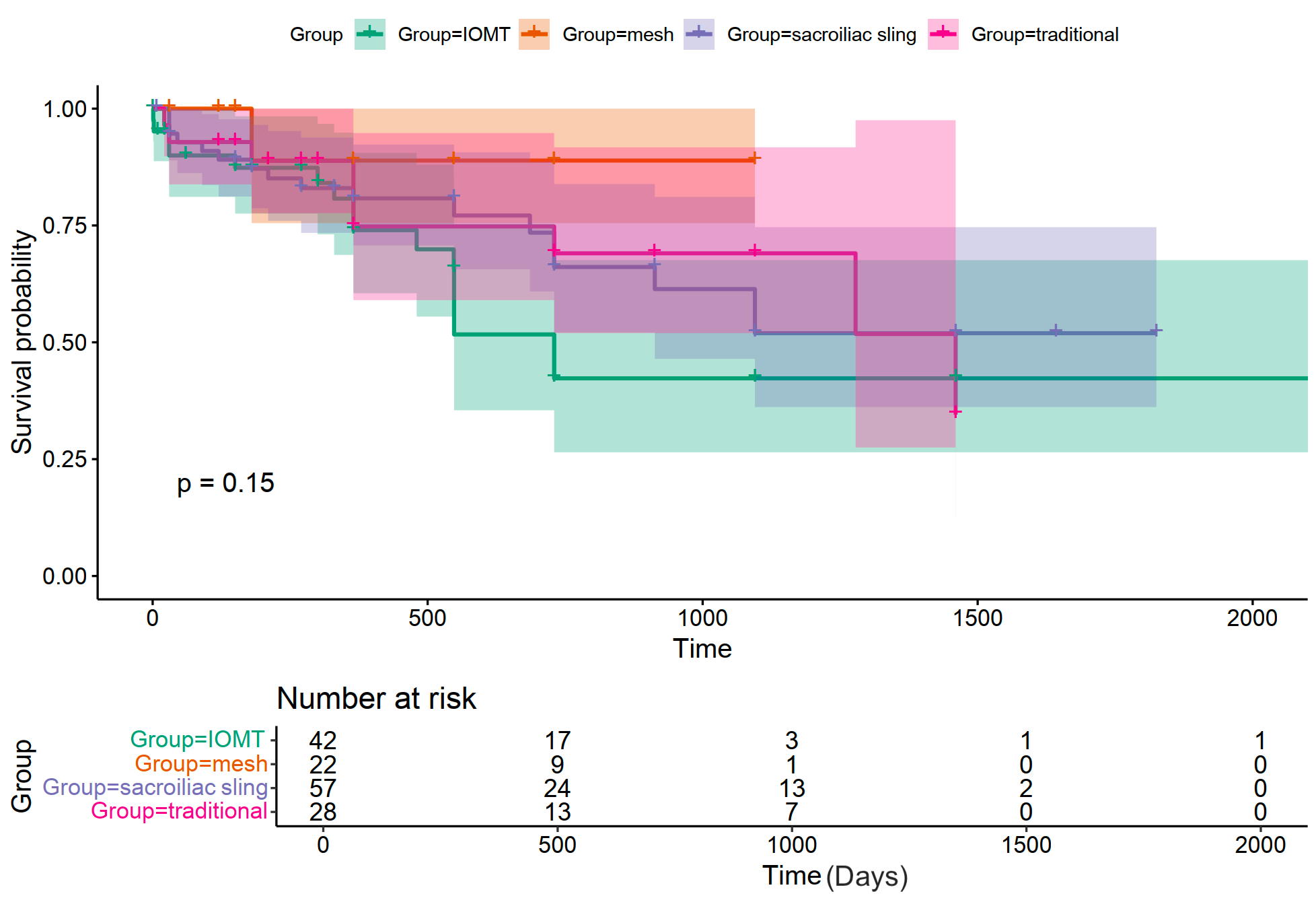

Supplement: Supplementary file 1 [file animals-15-01206-s001.zip › animals-3523538-supplementary/supplemetary meterial/Figure S1 The recurrence rate associated with surgical techniques.png]
